# Keap1-Knockdown Decreases Fasting-Induced Fatty Liver via Altered Lipid Metabolism and Decreased Fatty Acid Mobilization from Adipose Tissue

**DOI:** 10.1371/journal.pone.0079841

**Published:** 2013-11-04

**Authors:** Jialin Xu, Ajay C. Donepudi, Jamie E. Moscovitz, Angela L. Slitt

**Affiliations:** 1 Department of Biomedical and Pharmaceutical Sciences, University of Rhode Island, Kingston, Rhode Island, United States of America; 2 Institute of Biochemistry and Molecular Biology, College of Life and Health Sciences, Northeastern University, Shenyang, P. R. China; Nihon University School of Medicine, Japan

## Abstract

**Aims:**

The purpose of this study was to determine whether Nrf2 activation, via Keap1-knockdown (Keap1-KD), regulates lipid metabolism and mobilization induced by food deprivation (e.g. fasting).

**Methods and Results:**

Male C57BL/6 (WT) and Keap1-KD mice were either fed *ad* *libitum* or food deprived for 24 hours. After fasting, WT mice exhibited a marked increase in hepatic lipid accumulation, but Keap1-KD mice had an attenuated increase of lipid accumulation, along with reduced expression of lipogenic genes (acetyl-coA carboxylase, stearoyl-CoA desaturase-1, and fatty acid synthase) and reduced expression of genes related to fatty acid transport, such as fatty acid translocase/CD36 (CD36) and Fatty acid transport protein (FATP) 2, which may attribute to the reduced induction of Peroxisome proliferator-activated receptor (Ppar) α signaling in the liver. Additionally, enhanced Nrf2 activity by Keap1-KD increased AMP-activated protein kinase (AMPK) phosphorylation in liver. In white adipose tissue, enhanced Nrf2 activity did not change the lipolysis rate by fasting, but reduced expression of fatty acid transporters — CD36 and FATP1, via a PPARα-dependent mechanism, which impaired fatty acid transport from white adipose tissue to periphery circulation system, and resulted in increased white adipose tissue fatty acid content. Moreover, enhanced Nrf2 activity increased glucose tolerance and Akt phosphorylation levels upon insulin administration, suggesting Nrf2 signaling pathway plays a key role in regulating insulin signaling and enhanced insulin sensitivity in skeletal muscle.

**Conclusion:**

Enhanced Nrf2 activity via Keap1-KD decreased fasting-induced steatosis, pointing to an important function of Nrf2 on lipid metabolism under the condition of nutrient deprivation.

## Introduction

The reported overall prevalence of obesity among adults increased to 26.7% in 2009 from the Centers for Disease Control and Prevention [1]. Obesity-associated insulin resistance is a pathogenic event responsible for metabolic syndrome, which comprises Type 2 Diabetes (T2D), atherosclerosis, coronary heart disease and cancer, resulting in increased morbidity and mortality. Weight loss is well known as a useful strategy to prevent obesity in overweight or obese person with T2D. It is controversial whether weight loss is beneficial for reducing morbidity and mortality in individuals with high risk of obesity and diabetes [2,3], but a decrease in body weight is beneficial for the conversation of muscle energy and activity, which is important for enhancing the quality of life. Food deprivation/fasting or calorie restriction has been demonstrated as a useful and efficient strategy to control body weight.

Peroxisome proliferator activated receptor (Ppar) α signaling mediates the response to fasting in mice [4]. Pparα is expressed in tissues that have high rates of fatty acid oxidation, including liver, kidney, heart, and brown adipose tissue [5]; as well, white adipose tissue (WAT) [6]. Prolonged food deprivation induces a dramatic change of energy utilization and metabolism in mammalian model, via increasing the release of fatty acids from WAT, and enhancing fatty acid oxidation in liver [4]; this results in rapid body fat mobilization, and dramatically increases hepatic lipid accumulation [7]. Guan et al. reported that mice undergoing 24 hours of fasting exhibited excessive hepatic triglyceride (TG) accumulation and obvious hepatic steatosis, further suggesting that prolonged food deprivation induces dramatic energy mobilization and promotes fatty liver development process [8].

Hepatic steatosis, also known as fatty liver disease, is a reversible condition in which large vacuoles of TG or fat accumulate in hepatocytes, characterized by accumulation of lipid exceeding of 5% wet liver weight. Obesity, diabetes, and hyperlipidemia are the most common co-existing conditions frequently associated with fatty liver disease [9]. Upregulation of fatty acid uptake is associated with increased hepatic lipid accumulation, and results in hepatic steatosis [10]. Uptake of un-esterified long chain fatty acid into hepatocytes or adipocytes occurs through both a passive diffusion, and a saturable, protein-mediated mechanism, and is regulated by several membrane-associated proteins, including Fatty acid translocase/CD36 (CD36), Fatty acid transport protein (FATP) 1 through 6, and plasma membrane-associated fatty acid binding protein (FABPpm) [11]. Upregulation of CD36 was determined to be significantly associated with hepatic steatosis, hyperinsulinemia, and insulin resistance [12]. Deficiency of FATP2 or FATP5 genes reduced hepatic steatosis in mice [13,14], suggesting membrane-associated fatty acid transporter proteins play a significant role in the development of fatty liver.

Nuclear factor erythoid 2-related factor 2 (Nrf2, Nfe2l2) serves as a major regulator of a cellular defense system against oxidative stress, resides in the cytoplasm, and is sequestered by Kelch-like ECH-associated protein 1 (Keap1) for targeted degradation by Cullin 3-base E3 ubiquitin ligase [15]. Upon electrophilic/oxidative stress, the sequestration complex dissociates, Nrf2 escapes from Keap1-mediated repression and activates antioxidant-responsive element-dependent gene expression associated with the cytoprotective responses to a variety of drugs, toxicants, and cellular oxidative stresses [16]. It was reported that Keap1-knockdown (Keap1-KD, KD) mice exhibited enhanced Nrf2 signaling in liver [17]. However, the function of Nrf2 on lipid metabolism and fatty liver is not well understood and contradictory conclusions exist in the published literature. Enhanced Nrf2 activation (via Keap1-KD) attenuated methionine- and choline-deficient diet-induced fatty liver by increasing hepatic antioxidant and detoxification capacity [18]. A global analysis of mouse hepatic gene expression revealed that both genetic and pharmacologic activation of Nrf2 resulted in induction of a larger number of genes associated with lipid and glucose metabolism than xenobiotic detoxification, and tended to decrease lipid synthesis in the liver [19], suggesting that the presence of Nrf2 suppresses lipid metabolism. In contrast, Nrf2-null mice fed a chronic high fat-diet (HFD) exhibited less hepatic lipid accumulation and decreased hepatic steatosis, suggesting that presence of Nrf2 increases hepatic lipid accumulation and promotes the development of fatty liver in mouse model [20,21].

To reveal the effects of enhanced Nrf2 activity in lipid mobilization, WT and Keap1-KD mice were food-deprived for 24 hours to induce fatty liver, hepatic lipid accumulation and gene expression related to lipogenesis and fatty acid transport were determined. In livers of Keap1-KD mice, fasting-induced steatosis was ameliorated, along with decreased lipogenic gene expression and reduced induction of Pparα signaling, which subsequently reduced fatty acid transporter protein expression levels and the related fatty acid transport, indicating that enhanced Nrf2 activity via Keap1-KD prevented fasting-induced steatosis in mouse model.

## Materials and Methods

### Animals

Male 15- to 16-week-old C57BL/6 mice (WT) were purchased from Charles River Laboratories (Wilmington, MA). Keap1-KD mice were shared by Dr. Curtis Klaassen (University of Kansas Medical Center, Kansas City, KS) and Dr. Masayuki Yamamoto (Tohoku University Graduate School of Medicine, Sendai, Japan). Mice were fed with Teklad Rodent Diet 7012 (Harlan, Madison, WI) and allowed access to water *ad libitum* unless otherwise noted. All procedures were conducted in accordance with the NIH Guidelines for the Care and Use of Laboratory Animals and were approved by the University of Rhode Island Animal Care and Use Committee.

### Fasting Studies

Mice were divided into four groups: C57BL/6 fed *ad libitum* (WT-Fed), C57BL/6 food withheld (WT-Fasted), Keap1-KD fed *ad libitum* (KD-Fed), and Keap1-KD food withheld (KD-Fasted), n=8 to 9 for each group. The day before fasting, mice were housed singly and acclimated for one day. Next, mice were allowed access to water and food *ad libitum* or water only. Blood, liver, skeletal muscle (SKM) and the epididymal pad of WAT were collected 24 hrs after food was withheld. Blood was centrifuged at 5,000 x g for 15 mins at 4°C; serum was isolated and stored at -80°C. Two portions of the left lobe of each liver were ﬁxed in 10% formalin. Remaining liver was snap frozen in liquid nitrogen and stored at -80°C.

### Histopathology

Liver tissue was fixed in buffered formalin for 24 hrs, and then processed for paraffin embedding. Paraffin sections (5 μm) were cut and stained with hematoxylin and eosin (H&E). All procedures were performed according to typical histology protocols as performed by AML Laboratories (Baltimore, MD). For Oil Red O staining, frozen sections from liver were fixed with 10% formalin for 10 mins, then incubated with Oil Red O solution (six parts Oil Red O stock solution and four parts H_2_O; Oil Red O stock solution is 0.5% Oil Red O in 100% isopropanol) for 15 mins. Slides were counterstained with hematoxylin and mounted in glycerin jelly.

### Glucose Assay

Glucose levels in serum were analyzed using glucose assay kit (Cayman Chemical Company, Ann Arbor, MI) according to the manufacturer’s instruction. Five hundred microliters of glucose assay reagent was added to 5 µl of standard or serum sample, and then incubated at 37°C for 10 mins. The absorbance was measured at 500-520 nm by using a 96-well plate reader.

### Lipid Content Quantification

Liver or WAT (50 mg) were homogenized with PBS and extracted with mixture of chloroform-methanol (2:1; v/v). The residue was re-suspended in 1% Triton X-100 in 100% ethanol. TG and free fatty acid (FFA) content were determined by using TG (Pointe Scientific, Inc, MI,) and FFA (Wako Chemicals USA, Inc, VA) reagent kits. Serum cholesterol content was measured by using commercial cholesterol reagent kit (Pointe Scientific, Inc, MI).

###  Serum Analytes

Serum insulin, resistin, IL-6, TNFα, and glucagon concentration were determined by using a Millliplex MAP kit (Millipore, MA) according to manufacturer’s instruction. Serum glycerol content was determined by using a glycerol colorimetric assay kit (Cayman Chemical Company, Ann Arbor, MI). Serum β-hydroxybutyrate content was determined by using a commercial kit purchased from Pointe Scientific, Inc (Canton, MI).

### RNA Isolation and Quantitative Real-Time PCR

Total RNA was isolated by using TRIzol reagent (Invitrogen, CA) according to the manufacturer’s instruction. One microgram of total RNA was converted to single-stranded cDNA using oligo(d)T_18_ primers and mRNA levels were quantiﬁed by quantitative real-time PCR using Roche Lightcycler detection system (Roche Applied Science, Mannheim, Germany). Samples were run by using SYBR green and compared with levels of 18S rRNA or β-2 microglobulin as a reference housekeeping gene. PCR conditions were optimized for each gene using appropriate forward and reverse primers. The primers used are listed in Table S1. All oligonucleotides were synthesized by Invitrogen Inc., CA.

### Western Blot Analyses

The samples (40 µg) were subjected to SDS-PAGE on 10% polyacrylamide gels. Proteins were then electrophoretically transferred to PVDF membrane. After transferring, the membrane was blocked with 5% non-fat dry milk in TBS Tween-20 followed by incubation with primary antibodies overnight. The results were documented on X-ray ﬁlm by using an ECL detection kit (GE Healthcare Life Science, Piscataway, NJ). Primary antibodies were directed against: total-AMPKα, AMPKα phosphorylated at Thr172, total-AMPKβ1/2, AMPKβ1 phosphorylated at Ser108, Acc-1, Scd-1, total-Akt, Akt phosphorylated at Ser473 and GAPDH (Cell Signaling Technology, Danvers, MA); and Glut4 (from Sigma-Aldrich, St. Louis, MO).

### Primary Hepatocyte Culture

Mouse hepatocytes were isolated using a two-step perfusion as described previously [22]. Cells were isolated from 2-3 month-old male C57BL/6 and KD mouse, 1×10^6^ cells/well in 2 mL completed medium (MEM supplied with 10% FBS) were seeded on collagen-coated 6-well plates. After cells attachment (~4 hrs), they were cultured in serum-free MEM containing 1% ITS supplement (Invitrogen, CA). Twenty-four hours post plating, hepatocytes were harvested and the total protein was collected for the next measurement.

### VLDL Secretion Measurement

Male mice (8-week-old) fasted for 5 hrs were injected with Tyloxapol (Triton WR-1339, Sigma-Aldrich, St. Louis, MO) (0.5 mg/g body weight) in a 0.9% NaCl solution via tail vein. Blood samples were collected at 0, 60, and 120 mins after injection. Serum TG content was measured as before.

### Exogenous Lipid Clearance Rate Measurement

Male mice (8-week-old) were fasted overnight (>16 hrs) and then administered with olive oil via oral gavage (15 ml/kg body weight, Sigma-Aldrich, St. Louis, MO). Blood samples were collected at 0, 1, 3, and 6 hrs after oil administration. Serum was extracted and TG content was measured as before.

### GTT and ITT Assay

Glucose tolerance test (GTT) or insulin tolerance test (ITT) were performed by i.p. injecting glucose (1 g/kg) or insulin (1 U/kg) in WT and Keap1-KD mice fasted for 16 or 6 hrs. Blood glucose was determined by tail nick bleeds at 0, 30, 60, 90, 120, and 150 mins post glucose administration or 0, 15, 30, 60, and 120 mins post insulin administration.

### Acute Insulin Treatment in Mice

Acute insulin treatment was performed as described previously [23]. Male 15-week-old mice fasted overnight (>16 hrs) were anesthetized and a maximal bolus of insulin (5 U/kg body weight; Sigma-Aldrich, St. Louis, MO) was injected into the portal vein. Gastrocnemius muscles were collected 5 mins after the injection and immediately stored in liquid nitrogen. Protein extracts from the tissue samples were prepared and run on SDS-PAGE.

### Statistical Analysis

Quantitative data were presented as mean ± S.E. Statistic differences were determined by a one-way ANOVA followed by a Duncan’s Multiple Range *post hoc* test. All statistical tests with P<0.05 were considered as significant.

## Results

### Enhanced Nrf2 activity prevents fasting effects in Keap1-KD mice

**Figure 1:**
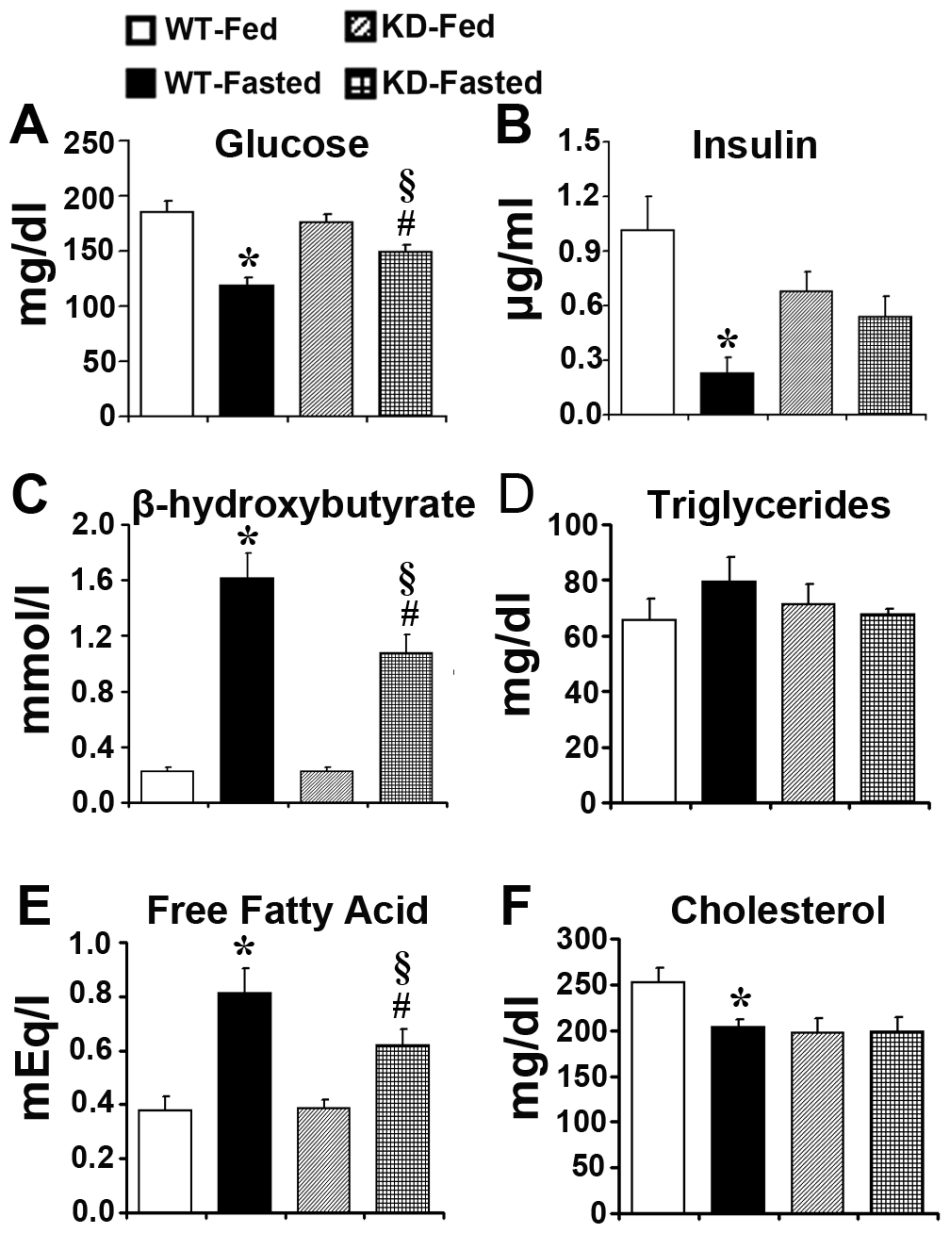
Enhanced Nrf2 activity prevents fasting effects in Keap1-KD mice. Serum content of (**A**) glucose, (**B**) insulin, (**C**) β-hydroxybutyrate, (**D**) TG, (**E**) FFA, and (**F**) cholesterol were determined in fed and fasted C57BL/6 (WT) and Keap1-KD (KD) mice (n=4-9 per group). *, P<0.05, WT-Fasted compared with WT-Fed mice; #, P<0.05, KD-Fasted compared with KD-Fed mice; §, P<0.05, KD-Fasted compared with WT-Fasted mice.

After fasting for 24 hrs, WT and Keap1-KD mice lost a substantial amount of total body weight (10.5% and 9.8%, respectively), liver weight (14.6% and 27.5%, respectively), and epididymal WAT weight (32.5% and 12.9%, respectively) (Table 1). Glucagon was not detectable in Keap1-KD mice in fed or fasted state. Resistin levels were deceased by 40.7% and 42.5%, and IL-6 levels were decreased by 66.1% and 62.1% in WT and Keap1-KD mice after fasting, respectively. TNFα levels were decreased to non-detectable concentrations after fasting in WT and Keap1-KD mice (Table S2). Serum glucose content was similar between WT and Keap1-KD mice in fed state. However, fasting reduced glucose content by 36.2% in WT mice, but only 15.2% in Keap1-KD mice (Figure 1A). Insulin content was decreased by 77.5% in WT mice, however only 20.8% in Keap1-KD mice (Figure 1B). β-hydroxybutyrate content was increased by 614% in WT mice after fasting, but only 380% induction was determined in Keap1-KD mice (Figure 1C), which suggested Keap1-KD interfered with fasting effects. Furthermore, serum TG content was similar among the four experimental groups (Figure 1D). FFA content was increased by 115% in WT mice, but only 60.6% increase was observed in Keap1-KD mice, and Keap1-KD mice exhibited lower FFA content compared with WT mice (Figure 1E). Cholesterol content was decreased in WT mice (by 19.5%), and no significant decrease was observed in Keap1-KD mice after fasting (Figure 1F).

**Table 1:** Body weight, liver weight and epididymal white adipose tissue weight of C57BL/6 and Keap1-KD mice after 24 hours fasting.

	**C57BL/6**		**Keap1-KD**	
	**Fed**	**Fasted**	**Fed**	**Fasted**
**Body Weight(BW), g**	27.70±0.46	24.79±0.66^*^	27.56±0.62	24.85±0.76^#^
**Liver Weight(LW), g**	1.23±0.04	1.05±0.03^*^	1.42±0.08^$^	1.03±0.04^#^
**LW/BW, %**	4.44±0.09	4.25±0.06	5.11±0.19^$^	4.15±0.13^#^
**Epididymal WAT Weight (EW), g**	0.40±0.07	0.27±0.06	0.85±0.17^$^	0.74±0.11^§^
**EW/BW, %**	1.39±0.27	1.09±0.23	2.80±0.51^$^	2.78±0.32^§^

Fifteen- to sixteen-week-old C57BL/6 (WT) and Keap1-KD (KD) mice were food withhold for 24 hrs, then were anesthetized and the organ weight was measured. * P<0.05, WT-Fasted compared with WT-Fed mice; $, P<0.05, KD-Fed compared with WT-Fed mice; # P<0.05, KD-Fasted compared with KD-Fed mice; § P<0.05, KD-Fasted compared with WT-Fasted mice.

### Enhanced Nrf2 activity prevents fasting-induced fatty liver in Keap1-KD mice

**Figure 2:**
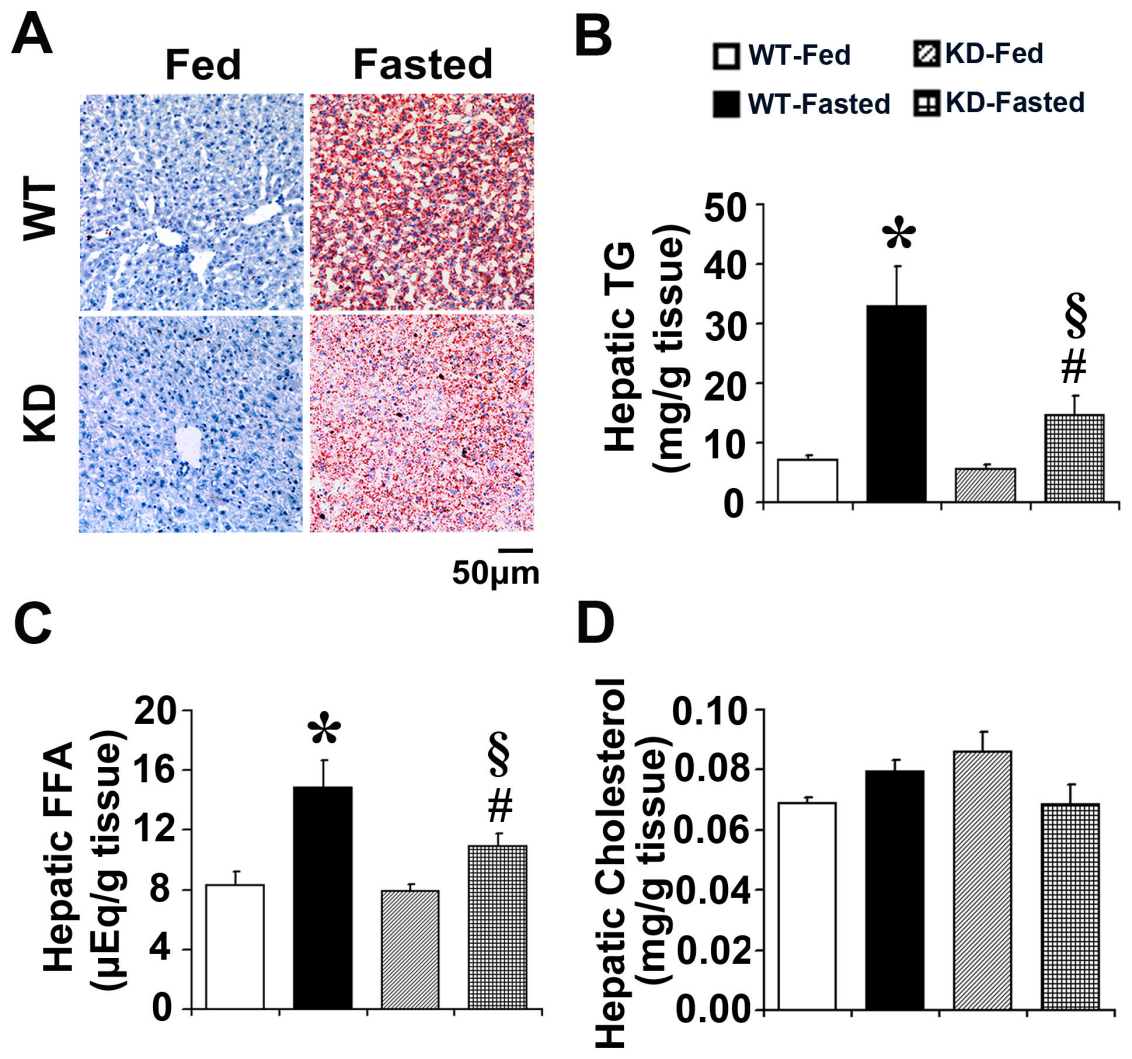
Enhanced Nrf2 activity prevents fasting-induced fatty liver in Keap1-KD mice. (**A**) Representative images of Oil Red O staining of livers sections in fed and fasted C57BL/6 (WT) and Keap1-KD (KD) mice. Magnification: 200×. Scale bar=50 μm (n=3 per group). Hepatic (**B**) TG**, **(**C**) FFA, and (**D**) cholesterol from fed and fasted C57BL/6 (WT) and Keap1-KD (KD) mice (n=4-6 per group). *, P<0.05, WT-Fasted compared with WT-Fed mice; #, P<0.05, KD-Fasted compared with KD-Fed mice; §, P<0.05, KD-Fasted compared with WT-Fasted mice.

No inflammation was observed in livers of fed or fasted WT and Keap1-KD mice (Figure S1). Oil Red O staining was performed to identify lipid content in liver. No obvious Oil Red O staining was observed in WT and Keap1-KD mice in fed state. The staining dramatically increased after fasting in WT mice, but the increase was attenuated in Keap1-KD mice, as noted by decreased Oil Red O staining in Keap1-KD mice compared with WT mice (Figure 2A). Lipids were also extracted from liver tissue and the content was quantified by spectrometer method. Hepatic TG and FFA content increased robustly in WT mice after fasting (362% and 79.7%, respectively), but the increase was attenuated in Keap1-KD mice (only 162% and 37.8%, respectively), suggesting enhanced Nrf2 activity prevented fasting-induced fatty liver (Figure 2B and 2C). In fed and fasted states, WT and Keap1-KD mice had comparable levels of hepatic cholesterol (Figure 2D). 

### Enhanced Nrf2 activity attenuates Pparα signaling induction and decreases lipogenic gene expression in liver by fasting

**Figure 3:**
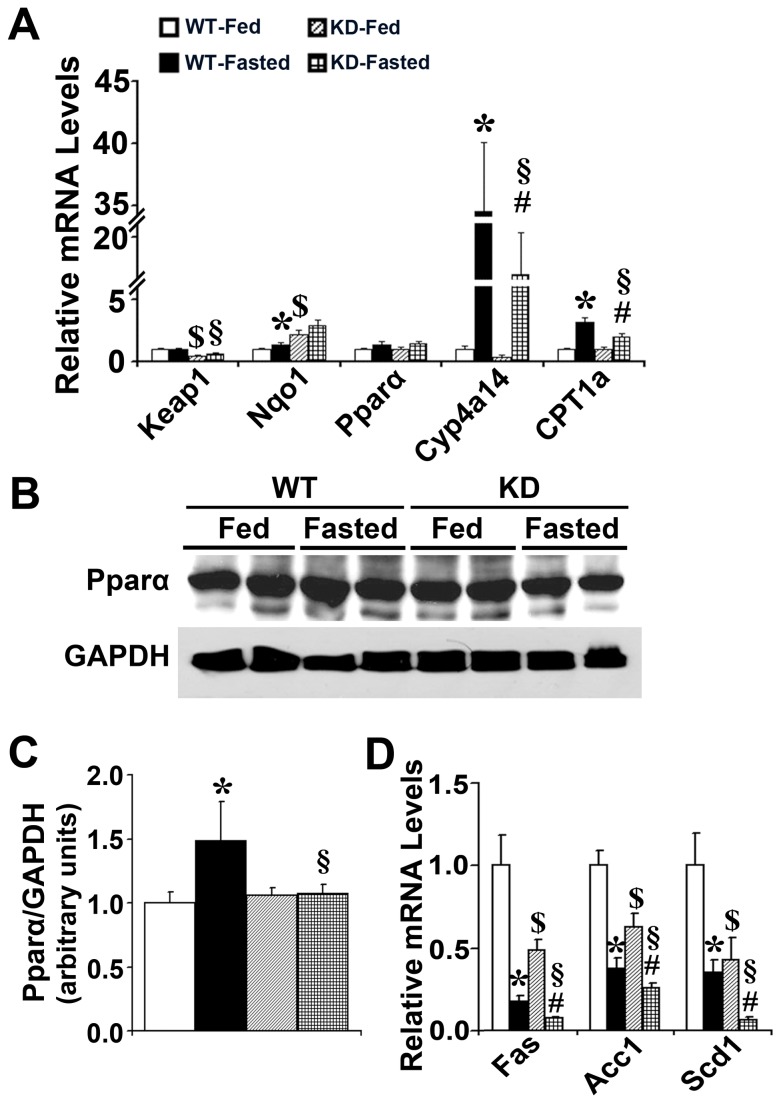
Enhanced Nrf2 activity attenuates Pparα signaling induction and decreases lipogenic gene expression in liver by fasting. (**A**) The induction of Cyp4a14 and CPT 1a by fasting was attenuated in liver of Keap1-KD (KD) mice. (**B**, **C**) Immunoblot analysis of Pparα in liver from fed or fasted C57BL/6 (WT) and Keap1-KD (KD) mice. (**D**)The expression of lipogenic genes, such as Fas, Acc1, and Scd1, was decreased in liver of Keap1-KD (KD) mice compared to C57BL/6 (WT) mice by fasting (n=4-6 per group). *, P<0.05, WT-Fasted compared with WT-Fed mice; #, P<0.05, KD-Fasted compared with KD-Fed mice; §, P<0.05, KD-Fasted compared with WT-Fasted mice.

Keap1 mRNA levels was decreased by 47.1% and 62.4% in fed and fasted Keap1-KD mice as compared with WT mice, respectively. NAD(P)H:quinone oxidoreductase 1 (Nqo1), a target gene of Nrf2 signaling [24], was induced in Keap1-KD mice, suggesting Nrf2 signaling activation in this genotype. No significant difference of Pparα mRNA levels was observed between WT and Keap1-KD mice in fed and fasted states. However, the expression of Cyp4a14 and Carnitine palmitoyltransferase (CPT) 1a, two target genes of Pparα signaling [25,26], were markedly induced in WT mice after fasting, but were attenuated in Keap1-KD mice. Furthermore, Pparα protein levels were induced by fasting in WT mice, but this induction was blocked completely in Keap1-KD mice (Figure 3B). The protein levels in Keap1-KD mice were significantly decreased compared with WT mice (Figure 3C), suggesting that enhanced Nrf2 activation (via Keap1-KD) may impair fasting-induced induction of Pparα signaling in Keap1-KD mice (Figure 3A). Lipogenic gene expression was determined by quantitative real-time PCR to explore Nrf2 function on *de novo* lipogenesis. Fatty acid synthase (Fas), Acetyl-CoA carboxylase (Acc) 1, and Stearoyl-CoA desaturase-1 (Scd1) were decreased by fasting in WT mice, and Keap1-KD mice exhibited an even greater decrease, suggesting enhanced Nrf2 activity promoted fasting-induced lipogenic gene reduction. The current study suggests that Keap1-KD may reduce lipogenesis and lipid accumulation by inhibiting lipogenic gene expression in liver (Figure 3D).

### Enhanced Nrf2 activity increases AMPKα phosphorylation in liver of Keap1-KD mice

**Figure 4:**
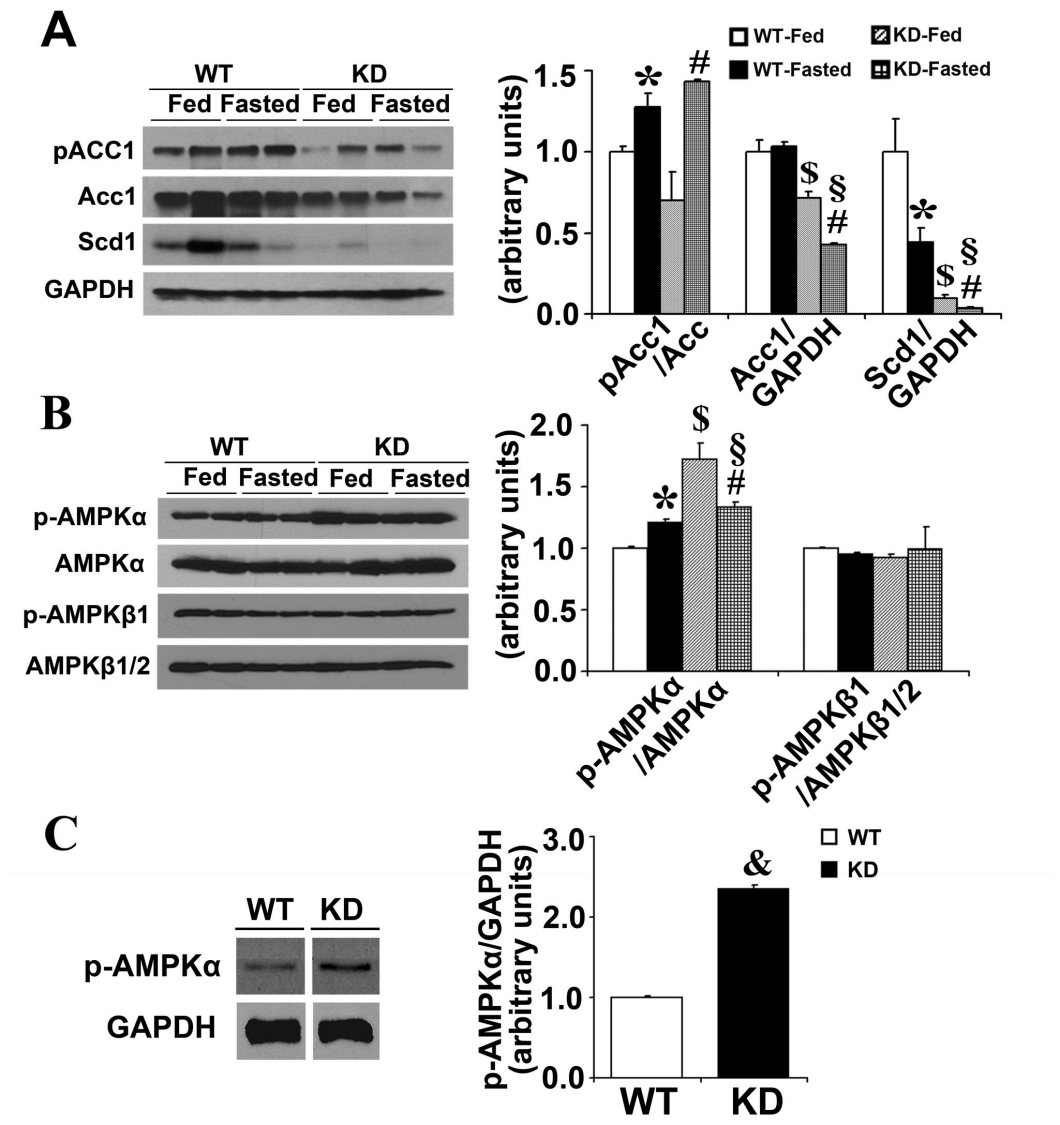
Enhanced Nrf2 activity increases AMPKα phosphorylation in liver of Keap1-KD mice. Immunoblot analysis of (**A**) Acc-1, Scd-1, and (**B**) total and p-AMPKα, total-AMPKβ1/2, and p-AMPKβ1 in livers of C57BL/6 (WT) and Keap1-KD (KD) mice. *, P<0.05, WT-Fasted compared with WT-Fed mice; #, P<0.05, KD-Fasted compared with KD-Fed mice; §, P<0.05, KD-Fasted compared with WT-Fasted mice. (**C**) p-AMPKα levels were increased in primary hepatocytes isolated from C57BL/6 (WT) and Keap1-KD (KD) mice. &, P<0.05, KD compared with WT mice hepatocytes.

Figure 4A illustrates that, from protein levels, the expression of hepatic lipogenic genes, Acc1 and Scd1, was decreased in Keap1-KD mice by fasting than WT mice, which is in agreement with the previous observation (Figure 3B). Phosphorylated-Acc was slightly increased in Keap1-KD mice than WT mice in fasted state (P=0.058). In order to explore the mechanism by which Nrf2 activation regulates lipogenic gene expression, AMPK signaling activation was evaluated by western blot. Fasting increased p-AMPKα levels in WT mice, which is also seen in the previous study [27]. Nrf2 significantly increased p-AMPKα levels in Keap1-KD mice in fed and fasted states than WT mice. There was no significant difference of p-AMPKβ1 and p-AMPKβ1/AMPKβ1/2 in fed and fasted WT and Keap1-KD mice (Figure 4B). Additionally, increased p-AMPKα levels were observed in primary hepatocytes isolated from Keap1-KD mice (Figure 4C), further suggesting Nrf2 activated AMPKα signaling, which may repress lipogenic gene expression (such as Acc1) in liver (Figure 3B).

### Enhanced Nrf2 activity decreases serum lipid clearance rate and VLDL secretion in Keap1-KD mice

**Figure 5:**
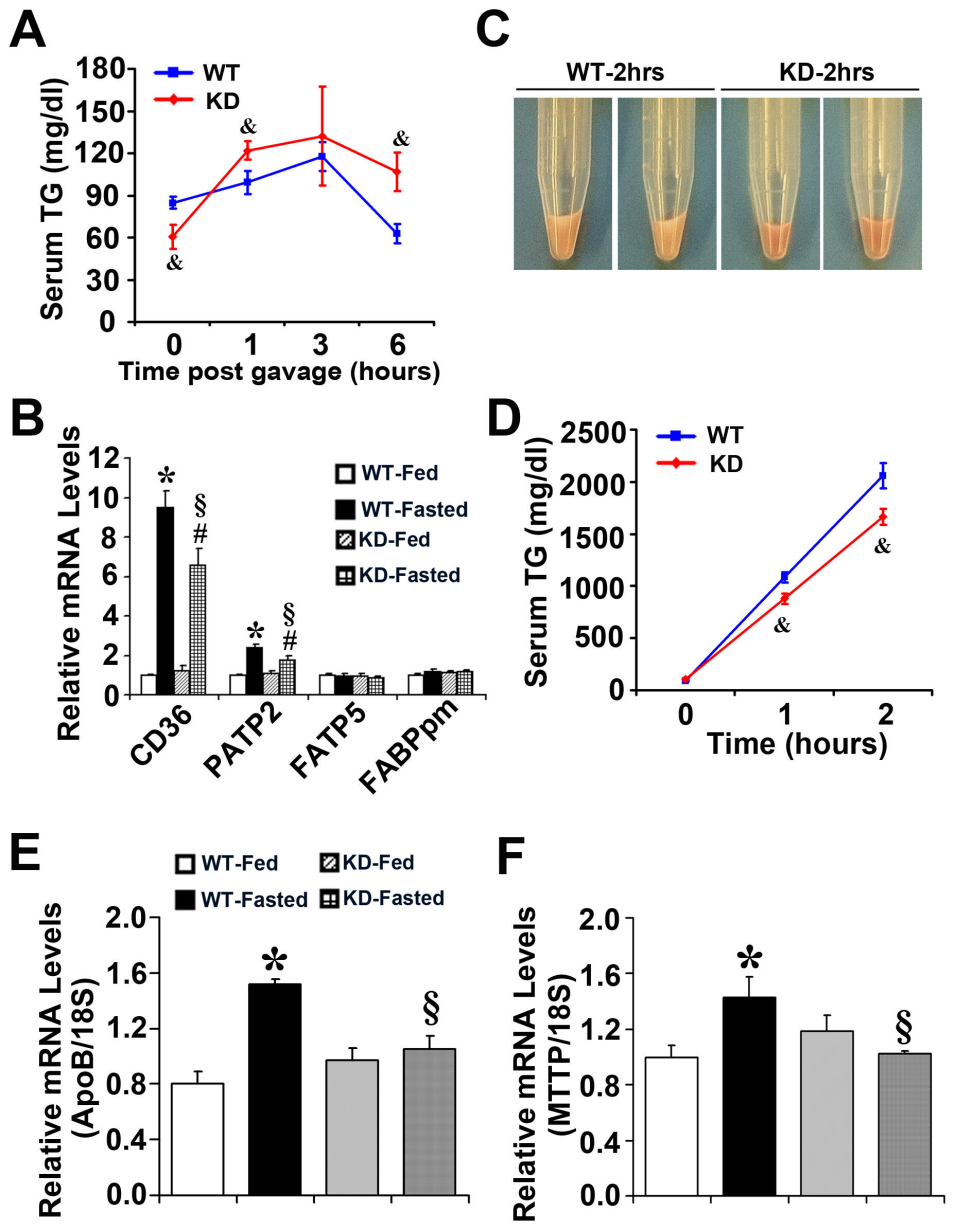
Enhanced Nrf2 activity decreases lipid clearance rate and VLDL secretion in Keap1-KD mice. (**A**) Enhanced Nrf2 activity impairs lipid clearance. Ten-week-old C57BL/6 (WT) and Keap1-KD (KD) mice were fasted overnight (more than 16 hrs), and then administered olive oil (15ml/kg) by oral gavage, serum TG content was measured 0, 1, 3, and 6 hrs after oil administration (n=5 to 8 per group) (&, P<0.05, Keap1-KD compared with WT mice). (**B**) The expression of fatty acid transporters of CD36 and FATP2 was deceased in liver from Keap1-KD mice compared with WT mice by fasting (n=4 to 6 per group). VLDL secretion was impaired in Keap1-KD mice (**C** and **D**). Ten-week-old C57BL/6 (WT) and Keap1-KD (KD) fasted for 5 hrs were injected with Tyloxapol (0.5 mg/g) and serum TG content was measured at 0, 1, and 2 hrs after injection (n=5 to 8 per group) (&, P<0.05, Keap1-KD compared with WT mice). The induction of (**E**) ApoB and (**F**) MTTP expression was attenuated in liver of Keap1-KD mice by fasting. The relative mRNA levels were quantified by quantitative real-time PCR and normalized with 18S as loading control (n=4-6 per group). *, P<0.05, WT-Fasted compared with WT-Fed mice; #, P<0.05, KD-Fasted compared with KD-Fed mice; §, P<0.05, KD-Fasted compared with WT-Fasted mice.

Lipid transport is associated with lipid accumulation [28]. In order to determine whether lipid transport alteration contributes to the observed reduction of hepatic lipid accumulation in Keap1-KD mice, WT and Keap1-KD mice were administered with exogenous lipids, and the lipid clearance rate was determined. Figure 5A illustrates that Keap1-KD mice exhibited higher content of serum TG than WT mice at 1 and 6 hrs after oil administration, even though these mice had lower TG content at time 0, suggesting enhanced Nrf2 activity decreased the exogenous lipid clearance rate. In order to explore which molecular mechanism was involved, the mRNA levels of hepatic CD36, FATP2, FATP5, and FABPpm, the membrane-associated transporter protein, were determined. CD36 and FATP2 mRNA levels were significantly induced in WT mice after fasting, but the induction was attenuated in Keap1-KD mice (Figure 5B). FATP5 and FABPpm mRNA levels were similar in WT and Keap1-KD mice in fed and fasted states (Figure 5B). The current data suggest that enhanced Nrf2 activity inhibited fatty acid transporter expression, which could explain the impaired fatty acid transport to the liver and reduced hepatic lipid accumulation. Additionally, hepatic VLDL/TG secretion was determined in fasted WT and Keap1-KD mice. Figure 5C and 5D illustrate that WT mice had higher hepatic VLDL secretion rate than Keap1-KD mice. Apolipoprotein B (ApoB) and Microsomal triglyceride transfer protein (MTTP), the two genes responsible for hepatic VLDL assembly and secretion, were significantly induced in WT mice by fasting, but the induction was attenuated in Keap1-KD mice, further suggesting enhanced Nrf2 activity decreased VLDL secretion from the liver (Figure 5E and 5F). Reduced VLDL secretion was associated with increased hepatic steatosis [29], but Keap1-KD mice exhibited decreased hepatic lipid content with repaired VLDL secretion. It is possible that this effect was overwhelmed by others, such as the attenuated hepatic lipogenesis or fatty acid uptake.

### Enhanced Nrf2 activity decreases fatty acid transport and results in increased fatty acids content in white adipose tissue

**Figure 6:**
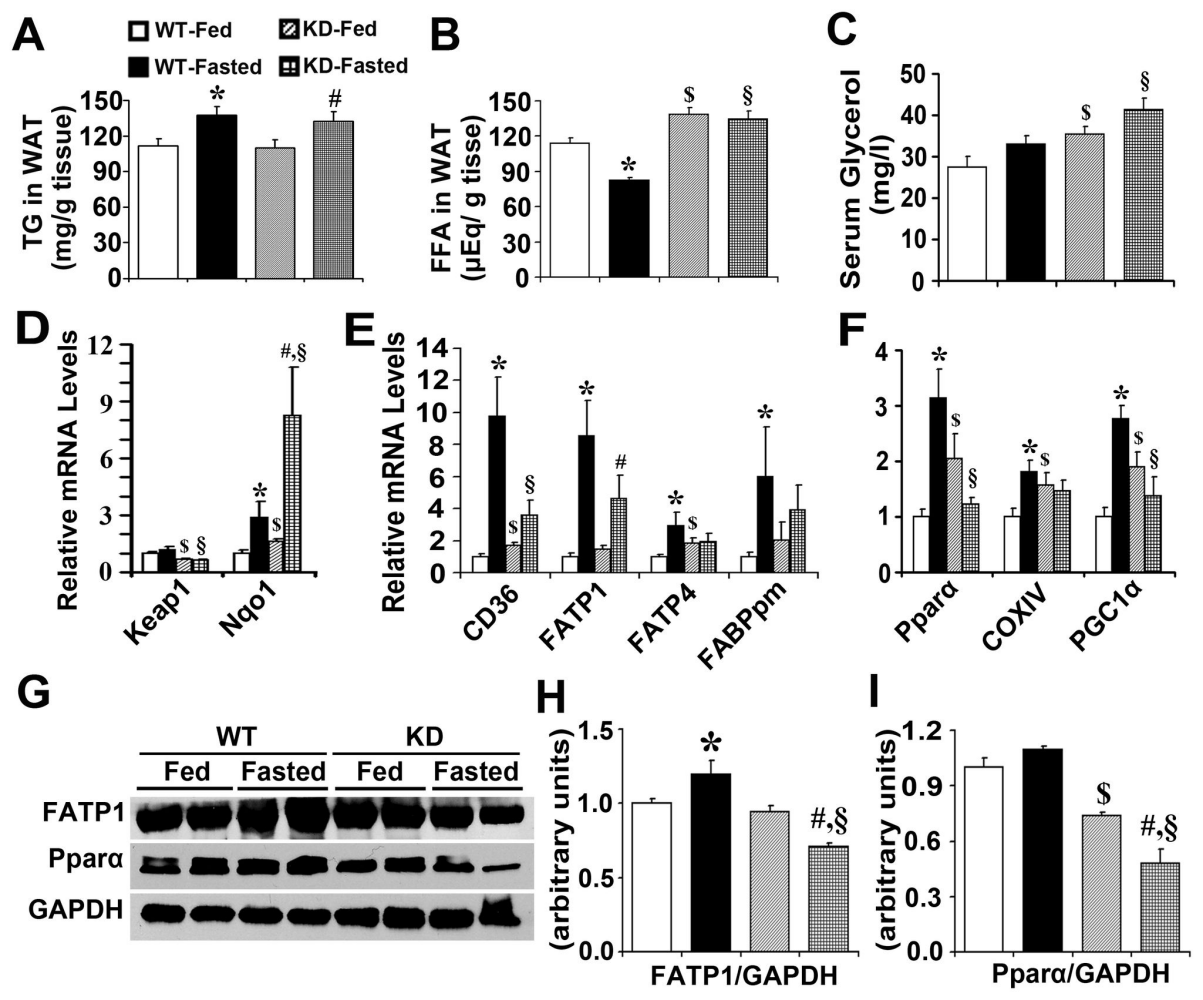
Enhanced Nrf2 activity decreases fatty acid transport and results in increased fatty acids content in white adipose tissue. (**A**) TG and (**B**) FFA content were measured in white adipose tissue of fed and fasted C57BL/6 (WT) and Keap1-KD (KD) mice (n=5-6 per group). (**C**) Nrf2 increased serum glycerol contents in Keap1-KD mice. (**D**, **E**, **F**) The induction of Pparα signaling and fatty acid transporter expression by fasting was attenuated in white adipose tissue of Keap1-KD mice. Total RNA was isolated from fed or fasted C57BL/6 (WT) and Keap1-KD (KD) mice and the relative mRNA levels were quantified by quantitative real-time PCR and normalized with β-2 microglobulin as loading control (n=4-6 per group). (**G**, **H**, **I**) Immunoblot analysis of Pparα and FATP1 in white adipose tissue from fed or fasted C57BL/6 (WT) and Keap1-KD (KD) mice. *, P<0.05, WT-Fasted compared with WT-Fed mice; $, P<0.05, KD-Fed compared with WT-Fed mice; #, P<0.05, KD-Fasted compared with KD-Fed mice; §, P<0.05, KD-Fasted compared with WT-Fasted mice.

TG content in WAT was increased in WT and Keap1-KD mice, but no significant difference was observed between these two genotypes in fed and fasted states (Figure 6A). Food deprivation is known to decrease serum insulin content, which induces lipolysis in WAT, and increases release of FFA and glycerol to peripheral circulatory system [30]. FFA content was decreased by 28% in WAT from WT mice, but the decrease was completely blocked in Keap1-KD mice (Figure 6B). Fasting induced serum glycerol content by 20.0% and 17.2% in WT and Keap1-KD mice, respectively. However, Keap1-KD mice exhibited higher content of serum glycerol than WT mice in fed and fasted states (Figure 6C). No significant difference of lipase expression was observed in WT and Keap1-KD mice in fed and fasted states, excluding Adipose triglyceride lipase (ATGL), which was induced by fasting in WT mice (Figure S2). Fasting is known to increase lipolysis and FFA export from WAT, which reduces FFA content inside of adipocytes (Figure 6B). However, it was hypothesized that in Keap1-KD mice, enhanced Nrf2 activity may impair the fatty acid transport out of the WAT. Next, it was determined whether the expression of fatty acid transporter protein was modulated by Nrf2 activation. The mRNA levels of CD36, FATP1 and FATP4, and FABPpm, which are predominantly expressed in WAT, were determined by quantitative real-time PCR in WT and Keap1-KD mice. Keap1 mRNA levels were decreased in WAT of Keap1-KD mice (about 67.2% and 55.5% compared with WT mice in fed and fasted states, respectively), which was associated with increased Nqo1 mRNA levels (Figure 6D). CD36, FATP1, FATP4 and FABPpm mRNA levels were significantly induced in WAT of WT mice after fasting, but this induction was attenuated in Keap1-KD mice (Figure 6E), suggesting Nrf2 activation via Keap1-KD inhibited fatty acid transport related gene expression, perhaps impairing fatty acid transport out of WAT and resulting in increased lipid accumulation within the adipocytes. Fasting induced Pparα mRNA expression along with the target gene, cytochrome c oxidase subunit IV (COXIV) [31], but this induction was completely blocked in Keap1-KD mice, as denoted by the reduced expression of Pparα and COXIV. Additionally, induction of PGC1α, which plays an important role in mediating fasting effects [32], was completely blocked in Keap1-KD mice (Figure 6F). Furthermore, FATP1 protein levels were significantly induced by fasting in WT mice, with no significant difference in Pparα signaling observed between WT and Keap1-KD mice; however, there is a significant decrease in Keap1-KD mice compared with WT mice in fasted state (Figure 6G-I).

### Enhanced Nrf2 activity increases glucose tolerance and Akt phosphorylation in SKM upon insulin challenge

**Figure 7:**
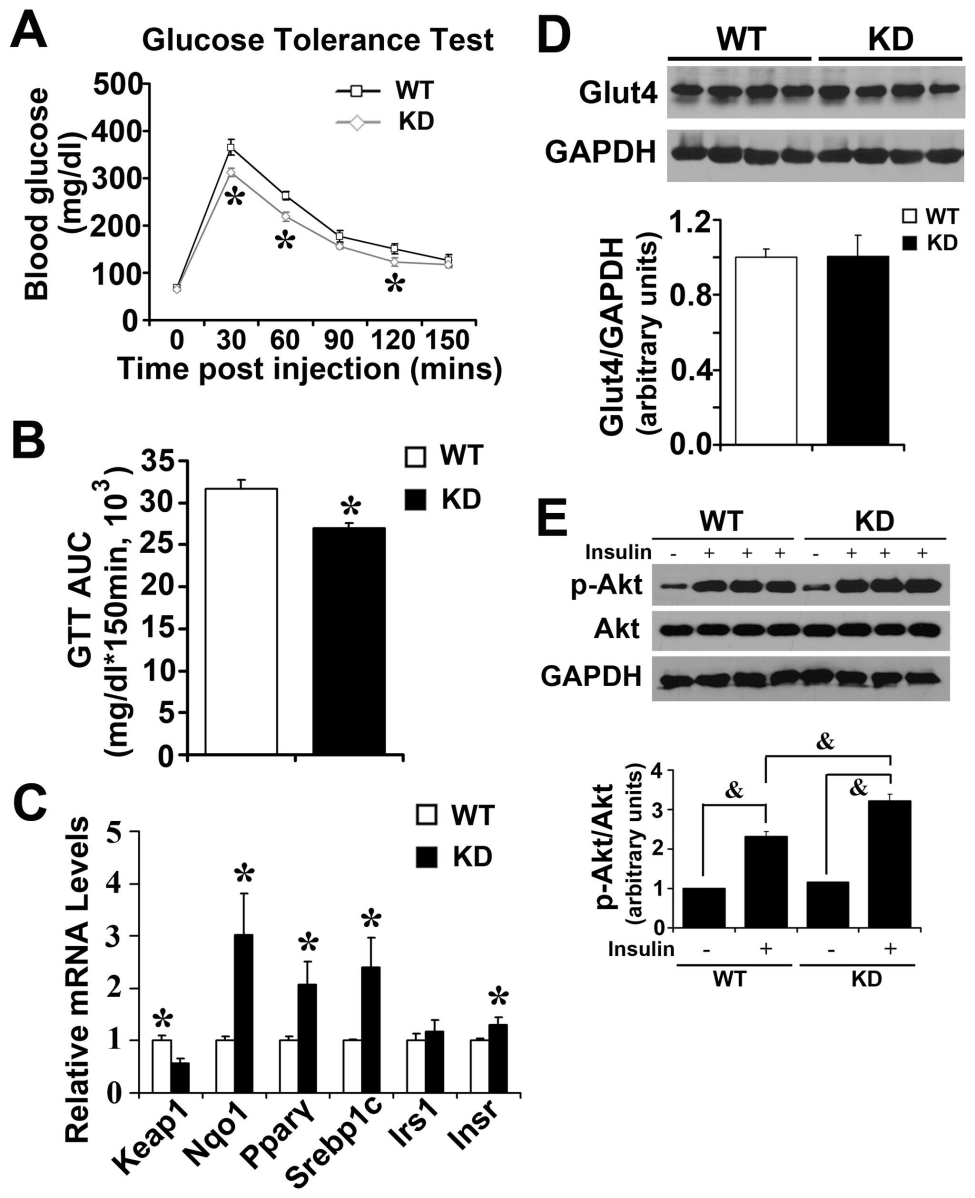
Enhanced Nrf2 activity increases glucose tolerance and Akt phosphorylation upon insulin challenge. (**A** and **B**) Keap1-KD mice have a lower glucose load. Ten-week-old C57BL/6 (WT) and Keap1-KD (KD) mice fasted overnight were challenge with glucose (2g/kg) and blood glucose levels were determined at 0, 30, 60, 90, 120, 150 mins after glucose administration (n=6 per group) (*, P<0.05, Keap1-KD compared with WT mice). (**C**) Gene expression in skeletal muscle from C57BL/6 (WT) and Keap1-KD (KD) at 16-week-old age. The relative mRNA levels were quantified by quantitative real-time PCR and normalized with 18S as loading control (n=5 to 6 per group) (*, P<0.05, Keap1-KD compared with WT mice). (**D**) Immunoblot analysis of Glut4 in skeletal muscle of C57BL/6 (WT) and Keap1-KD (KD) mice at 16-week-old age. GAPDH was used as loading control. (**E**) Enhanced Nrf2 activity increased p-Akt levels upon insulin administration in skeletal muscle. Fifteen-week-old C57BL/6 (WT) and Keap1-KD (KD) mice fasted for 6 hrs were injected with a maximal bolus of insulin (5 U/kg) via portal vein. Five minutes later, gastrocnemius muscles were removed and snap frozen in lipid nitrogen. &, P<0.05 as indicated.

Imbalance of fatty acid transport is associated with insulin resistance [33]. WT and Keap1-KD mice at normal physiological condition were challenged with glucose and insulin to detect insulin signaling status. Previously it was reported that aged Keap1-KD mice exhibited comparable glucose tolerance to corresponding C57BL/6 controls [34]. However, reports do not exist regarding whether there is any effect of Keap1-KD on glucose metabolism in young adult mice under normal physiological conditions. Figure 7A illustrates that Keap1-KD mice exhibited a lower glucose load than WT mice after acute glucose challenge, suggesting higher glucose tolerance in Keap1-KD mice. An AUC_GTT_ assay further confirmed this observation (Figure 7B). No difference of insulin tolerance was detected between WT and Keap1-KD mice (Figure S3). In SKM, Keap1 mRNA levels were decreased by 43% in Keap1-KD mice, and Nqo1 mRNA levels were increased, suggesting Nrf2 signaling was activated in Keap1-KD mice. The mRNA levels of Pparγ, Sterol regulatory element binding protein 1c (Srebp1c), and Insulin receptor (Insr), which are associated with insulin signaling, were increased in Keap1-KD mice, suggesting that enhanced Nrf2 activity increased insulin signaling in SKM of mice. No significant difference of Glut4 protein levels was detected from western blot (Figure 7D). Next, WT and Keap1-KD mice were challenged with insulin to explore the response to insulin by the modulation of enhanced Nrf2 activity. No difference of total-Akt was observed between WT and Keap1-KD mice, but the phosphorylated-Akt levels were increased in Keap1-KD mice (Figure 7E), suggesting enhanced Nrf2 activity increased insulin signaling in SKM upon insulin challenge.

## Discussion

The current study began with the observation that Keap1-KD mice exhibited a dramatic difference in their susceptibility to the stimulus of fasting – with increased serum glucose and insulin levels, reduced fasting-induced hepatic steatosis, and decreased epididymal WAT weight loss compared with controls (Table 1). Enhanced Nrf2 activity reduced fasting-mediated induction of Pparα signaling in liver and WAT, decreased hepatic *de novo* lipogenic gene expression of Fas, Acc1 and Scd1, and reduced gene expression related to fatty acid transport in liver and WAT. Enhanced Nrf2 activity promoted glucose tolerance via increased Akt phosphorylation levels, and increased the response upon insulin administration in SKM. It was reported that pharmacologically-enhanced Nrf2 activity by Oltipraz prevented insulin resistance and attenuated the HFD effect on glucose disposal, and reduced HFD-induced obesity [35], further demonstrated that Nrf2 played negative roles in the regulation of lipid metabolism.

Genetically-enhanced Nrf2 activity by Keap1-KD prevented fasting-induced hepatic steatosis in mouse liver. Contradictory reports exist regarding the role of Nrf2 function on regulation of hepatic steatosis. Post 5 days of methionine- and choline-deficient diet, WT mice showed higher hepatic lipid accumulation as evidence by the increased hepatic fatty acid uptake and reduced VLDL secretion [29]. Nrf2-null mice exhibited more severe hepatic steatosis, while Keap1-KD mice exhibited less, along with decreased CD36 and Fgf21 expression, suggesting Nrf2 negatively regulate lipogenesis and hepatic lipid accumulation [18]. Moreover, mice administered with oleanolic triterpenoid 1-[2-cyano-3,12-dioxooleana-1,9(11)-dien-28-oyl] imidazole (CDDO-Im), a potent Nrf2 activator, for 21 or 95 days, displayed protection against HFD-induced body weight gain and WAT mass increasing, along with decreased hepatic lipid accumulation. However, this observation was not observed in Nrf2-null mice, suggesting CDDO-Im prevented obesity and hepatic lipid accumulation via an Nrf2-dependent mechanism [36]. Additionally, upon 4 weeks of HFD-treatment, Nrf2-null mice exhibited increased expression of lipogenic genes and transcription factors, such as Srebp1c and 2, Fas, and Acc1, and increased hepatic FFA content, further demonstrated that Nrf2 negatively regulate hepatic lipid accumulation [37]. It was noted that the research work from Pi et al. [21] and Chartoumpekis et al. [38], which reported that Nrf2-null mice were somehow protected against HFD-induced obesity and fatty liver for long-term treatment (12 and 24 weeks, respectively). Our previous work reported Keap1-KD mice had less lipid accumulation in WAT upon short-term HFD-treatment (5 weeks) [24], and exhibited higher hepatic lipid accumulation for long-term treatment (24 weeks) [34], suggesting Nrf2 may play dual roles in regulating lipid accumulation regarding different durations of high lipid loading. In the different models explored, the interactions between diet (low or high fat diet), length of time on diet, and intact hormonal axis (leptin presence) must be considered. In models of Nrf2 activation, consideration must be given to off-target effects that could be beneficial or perhaps interact with the Nrf2 pathway and Keap1. Overall, for future studies, an integrate body of work that compares WT, Keap1-KD, and Nrf2-null mice treated with short- and long-term HFD-intervention should be considered to help advance the field.

Hepatic lipid accumulation is derived from: 1) *de novo* lipid synthesis from fatty acids in the liver (lipogenesis); 2) uptake of circulating albumin-bound fatty acids; and 3) uptake of circulating VLDL and chylomicron lipid particles [39]. Fasting is described to decrease lipogenesis and inhibit lipogenic gene expression of Srebp1c, Acc1, and Fas in liver [40]. Decreased expression of Fas, Acc1, and Scd1 have been reported in association with reduced hepatic lipid accumulation in mouse liver [37]. The current study demonstrated that increased Nrf2 activity in Keap1-KD mice decreased lipogenic gene expression of Fas, Acc1, and Scd1 (Figure 3), which contributed to the decreased fasting-induced hepatic steatosis and lipid accumulation.

Another likely source of excess lipid for hepatic steatosis is the increased influx of FFAs. It has been reported that various membrane-associated fatty acid-binding proteins were associated with the transport of cellular fatty acids in intestine, liver, WAT, SKM and heart. The most characterized of these are CD36, long-chain fatty acyl-CoA synthetases, and fatty acid transport proteins 1 through 6 [11]. CD36 is ubiquitously expressed in liver, heart, adipose tissue, and intestine [41]. FATP1 is the most prominent one in WAT, FATP2 is almost exclusively expressed in liver and kidney [11]. Expression of FATP4 was increased in human adipose tissue with obesity [42]. Expression of FATP5 was reported in liver exclusively [43]. FABPpm protein has been identified in liver [44], and WAT [45]. Hepatic steatosis is associated with enhanced expression of CD36 in patients with non-alcoholic steatohepatitis [12]. Also, hepatic steatosis induced by HFD significantly increased CD36 expression, and forced expression of hepatic CD36 in lean mice was reported to increase hepatic fatty acid uptake and TG storage, further supporting the observation of hepatic exiting in diet-induced-obesity mice [46]. It was reported that FATP2 overexpression increased fatty acids uptake in human hepatoma cells, suggesting FATP2 may play a key role in regulating fatty acid uptake and subsequent cellular lipid accumulation [47]. There is no significant difference in FATP5 or FABPpm expression between WT-fasted and KD-fasted groups, a possible reason being that these two genes are expressed to a relatively lesser degree than CD36 and FATP2 in liver.

It has been well summarized that PPAR/RXR signaling pathway regulates FATP expression and hepatic lipid accumulation [48]. Studies in mice demonstrated that Pparα signaling regulated the gene expression related to lipid metabolism in liver, including mitochondria and peroxisomal fatty acid oxidation, fatty acid uptake, utilization and catabolism, lipoprotein assembly and transport [49–51]. Fibrates, known as Pparα activators, induce FATP mRNA levels and increase fatty acid uptake into liver [52]. Additionally, Wy14,643 (Pparα activator), induced fatty acid transport related genes, but the induction was blocked in Pparα-null mice, suggesting the induction was via a Pparα-dependent mechanism [53]. In the current study, enhanced Nrf2 activity impaired the fasting effects, and decreased Pparα signaling activation, described by the attenuated induction of Pparα protein levels, and the two target genes of Cyp4a14 and Cpt 1a (Figure 3A), contributing to decreased gene expression related to fatty acid transport and impair fatty acid influx to the liver in Keap1-KD mice.

During fasting, serum glucose and insulin levels decrease and glucagon levels increase, which results in an enhanced WAT lipolysis rate to stimulate the breakdown of fat stored in adipose tissue and subsequent release of fatty acids and glycerol as fuel. Glycerol content increased similarly in WT and Keap1-KD mice by fasting (Figure 6C), but was higher in Keap1-KD mice than WT mice in fed and fasted states, respectively. A possible reason is that Keap1-KD mice have a greater amount of WAT than WT mice, which would increase the lipolysis rate for the tissue and release more FFA into the blood (Table 1). There is an increase of TG content in WAT with fasting in WT and KD mice, which is consistent with our previous work and the published literature [24] [54], the possible reason is that fasting remove water from the tissue, which will increase the relative weight of lipid content. But there is no significant difference of TG content between these two phenotypes after fasting, this may be due to the comparable expression levels of lipase, such as Hormone-sensitive lipase (Hsl), ATGL, and Monoacylglycerol Lipase (MGL), which are important for TG breakdown in WAT (Figure S2). Food deprivation induced Pparα signaling, accompanied by increased PGC1α expression (Figure 6F), followed by enhanced expression of genes related to fatty acid transport, such as CD36, FATP1, FATP4, and FABPpm in WT mice, which reduced lipid content inside of WAT (Figure 6B). However, the expression of membrane-associated protein responsible for fatty acid transport was attenuated in Keap1-KD mice, which may impair fatty acid transport outside of adipocytes, resulting in enhanced fatty acids content inside of WAT. 

It was noted that enhanced Nrf2 activity induced AMPK activation in the liver (Figure 4). AMPK exists as a heterotrimeric complex consisting of a catalytic subunit α and two regulatory subunits β and γ involved in heterotrimer formation and ligand sensing [55]. The conversational activity of serine/threonine kinase activity of AMPK is supported by α subunit. AMPK has been identified as a key regulator of energy status and play key role in protecting against energy-restricted conditions, such as food deprivation and caloric restriction in the liver [56]. It has been demonstrated that during fasting-feeding transition, AMPK is activated in the liver and contributes to the switch between lipogenesis and β-oxidation as a result of its energy sensor function [57]. Acc1 is an important rate-controlling enzyme for the synthesis of malonyl-CoA, which is important for *de novo* fatty acid synthesis in the liver. Inhibition of Acc1 by AMPK leads to a fall in malonyl-CoA content and a subsequent decrease in fatty acid synthesis, and results in decreased lipid accumulation in the liver. 

Glucose metabolism is closely related to lipid metabolism. Mice with impaired glucose tolerance exhibited higher hepatic lipid accumulation and more severe hepatic steatosis [58]. Enhanced Nrf2 activity increased glucose tolerance and insulin sensitivity (Figure 7). It was reported previously that Keap1-KD mice had comparable glucose tolerance to WT mice, which is different from the current study. A possible reason is that older mice were used (27 weeks) than in the current study (12 weeks). In addition, the previous study failed to use a sample size large enough to have sufficient statistical power (4~5 vs 8~9) [34]. Pparγ and Srebp1c, which are important transcription factor that mediated insulin function on glucose transport [59,60], were increased in Keap1-KD mice. Thiazolidinediones, known as Pparγ activators, are typically used as insulin sensitizers and therapeutically used in the treatment of T2D [61]. Insulin increased the mRNA levels of Glut4 by enhancing Srebp1c mRNA levels and the recruitment of Srebp1c to the putative sterol-response element in Glut4 promoter [62]. Glut4 is the membrane-associated protein and a major mediator of glucose removal from circulation to cells [63]. No significant difference of Glut4 protein content was observed between WT and Keap1-KD mice, suggesting the increased glucose tolerance in Keap1-KD mice may be not only determined by the modulation of Glut4 expression, but Glut4 translocation may play important roles in the current study. Activation of insulin signaling induced Akt phosphorylation, which promotes Glut4 translocation from cell surface to plasma membrane and increases glucose transport. In the current study, Keap1-KD mice exhibited higher p-Akt upon insulin administration, suggesting that Nrf2 promotes the insulin response, and finally enhances Glut4 translocation to facilitate glucose transport into cells. An ITT shows the ability of insulin-induced glucose removal, thus it was surprising that there was no difference in ITT between WT and KD mice. Our unpublished data demonstrated that enhanced Nrf2 activity by Keap1-KD can increase glucose production, suggesting this could compensate for the removal of glucose upon insulin administration in Keap1-KD mice.

Overall, the current study reports enhanced Nrf2 activity, genetically via Keap1-KD, prevented fasting-induced fatty liver by inhibiting fatty acid transport and impairing activation of the Pparα signaling pathway in liver. Enhanced Nrf2 activity did not change the lipolysis rate in WAT, but inhibited gene expression related to fatty acid transport, which impaired fatty acid transportation from WAT and increased fatty acid content inside of the tissue. Also, enhanced Nrf2 activity increased AMPK phosphorylation, which will inhibit the lipid synthesis in the liver. Lastly, the current study reported that enhanced Nrf2 activity increased response upon insulin administration and glucose tolerance ability. Overall, the Nrf2-Keap1 pathway has a role in regulating lipid metabolism during fasting.

## Supporting Information

Figure S1 **Representative pictures of hematoxylin and eosin staining of livers sections from C57BL/6 and Keap1-KD upon fasting.** C57BL/6 (WT) and Keap1-KD (KD) mice fasted for 24 hrs. Part of liver tissue was fixed with 10% formalin. Liver sections were cut (5 μm) and hematoxylin and eosin staining was performed. Scale bar = 100 μm.
(TIF)

Figure S2 **Expression of lipase in white adipose tissue of fed and fasted C57BL/6 and Keap1-KD mice.** C57BL/6 (WT) and Keap1-KD (KD) mice fasted for 24 hrs. Epididymal WAT was harvested and total RNA was extracted by Trizol. The target genes mRNA levels were determined by quantitative real-time PCR. The relative mRNA levels has been normalized with β-2 microglobulin. *, P<0.05, WT-fed vs. WT-fasted.
(TIF)

Figure S3 **Insulin tolerance test on C57BL/6 and Keap1-KD mice.** Twenty-week-old C57BL/6 (WT) and Keap1-KD (KD) fasted for 6 hrs were injected with insulin (1U/kg body weight) intraperitoneally. Blood glucose was determined by tails bleeds at 0, 15, 30, 60, and 120 mins post insulin administration.
(TIF)

Table S1 **List of primers sequence used.**
(DOC)

Table S2 **Serum cytokine content in C57BL/6 and Keap1-KD mice after 24 hours fasting.**
(DOCX)
