# Supplementary figures and images for: Keap1-Knockdown Decreases Fasting-Induced Fatty Liver via Altered Lipid Metabolism and Decreased Fatty Acid Mobilization from Adipose Tissue
Source: PLoS One. 2013 Nov 4;8(11):e79841. doi: 10.1371/journal.pone.0079841 (PMC3817107; doi:10.1371/journal.pone.0079841)

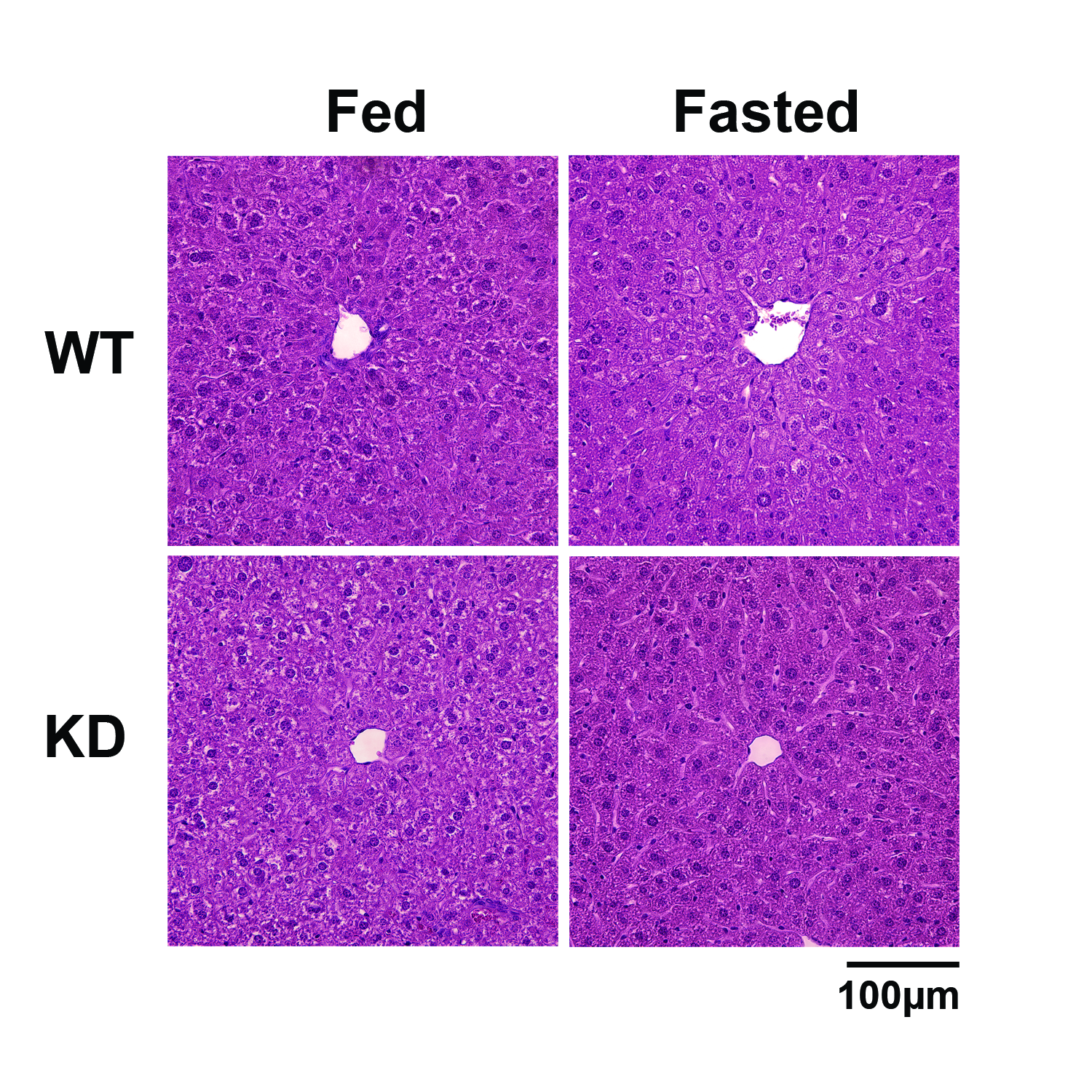

Supplement: Figure S1 — Representative pictures of hematoxylin and eosin staining of livers sections from C57BL/6 and Keap1-KD upon fasting. C57BL/6 (WT) and Keap1-KD (KD) mice fasted for 24 hrs. Part of liver tissue was fixed with 10% formalin. Liver sections were cut (5 μm) and hematoxylin and eosin staining was performed. Scale bar = 100 μm. (TIF) [file pone.0079841.s001.tif]

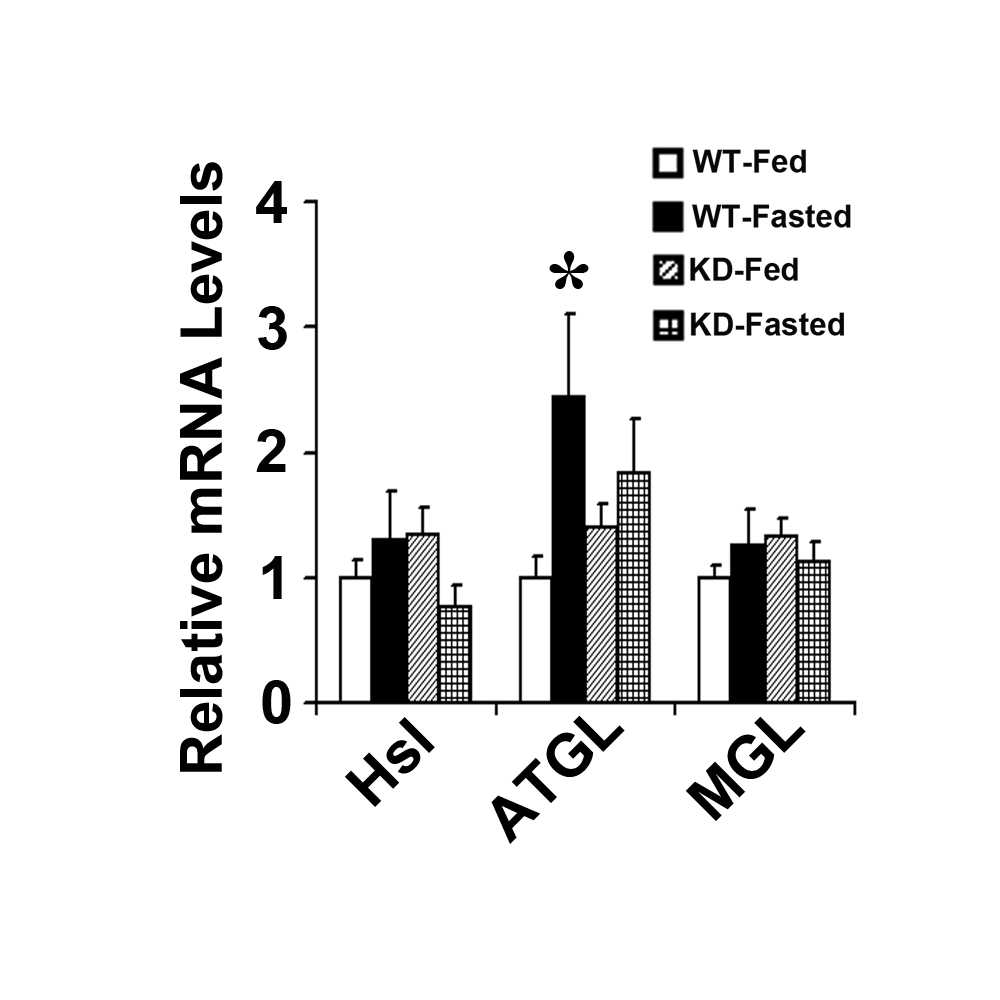

Supplement: Figure S2 — Expression of lipase in white adipose tissue of fed and fasted C57BL/6 and Keap1-KD mice. C57BL/6 (WT) and Keap1-KD (KD) mice fasted for 24 hrs. Epididymal WAT was harvested and total RNA was extracted by Trizol. The target genes mRNA levels were determined by quantitative real-time PCR. The relative mRNA levels has been normalized with β-2 microglobulin. *, P<0.05, WT-fed vs. WT-fasted. (TIF) [file pone.0079841.s002.tif]

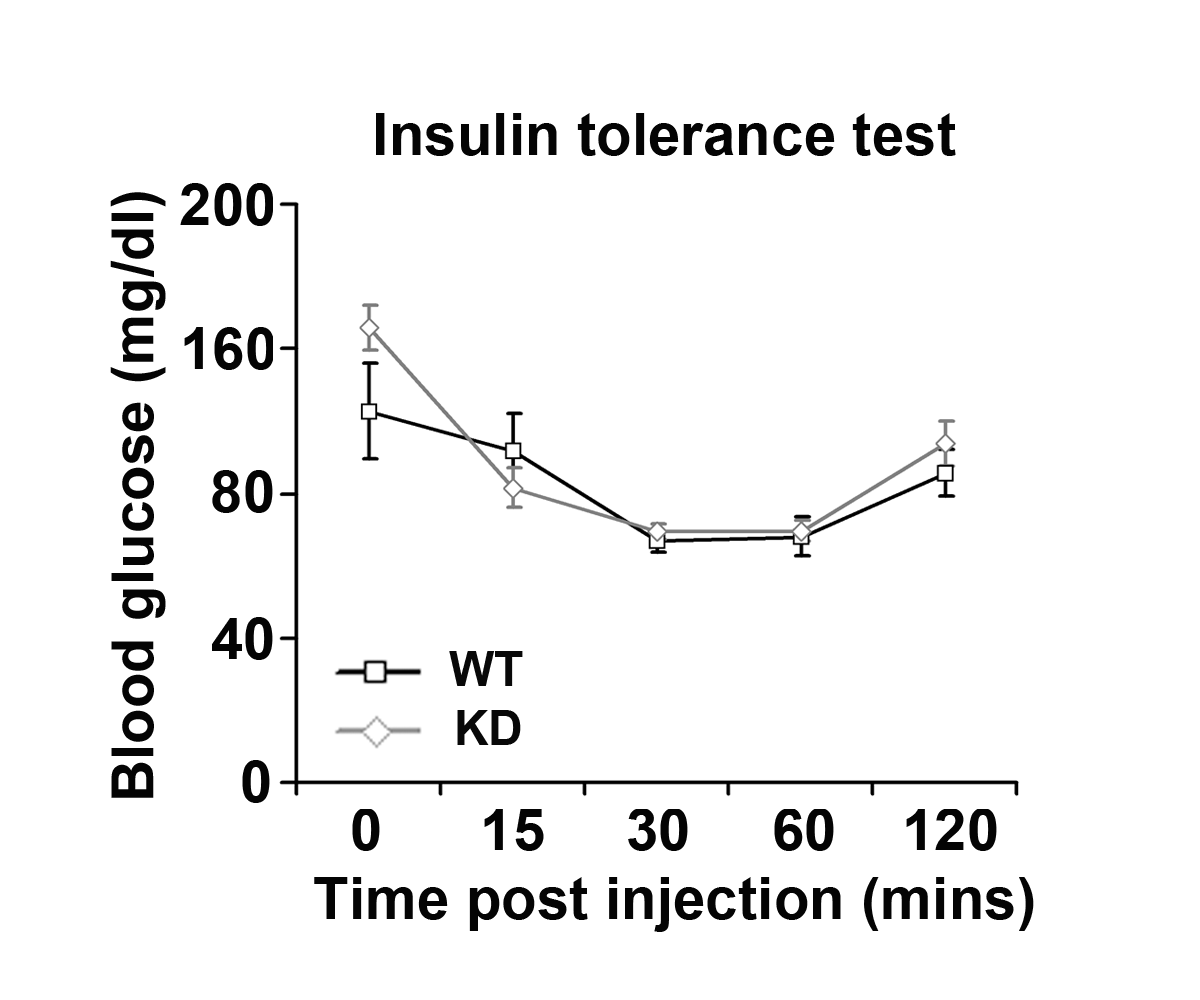

Supplement: Figure S3 — Insulin tolerance test on C57BL/6 and Keap1-KD mice. Twenty-week-old C57BL/6 (WT) and Keap1-KD (KD) fasted for 6 hrs were injected with insulin (1U/kg body weight) intraperitoneally. Blood glucose was determined by tails bleeds at 0, 15, 30, 60, and 120 mins post insulin administration. (TIF) [file pone.0079841.s003.tif]
