# Supplementary material for: Keap1-Knockdown Decreases Fasting-Induced Fatty Liver via Altered Lipid Metabolism and Decreased Fatty Acid Mobilization from Adipose Tissue
Source: PLoS One. 2013 Nov 4;8(11):e79841. doi: 10.1371/journal.pone.0079841 (PMC3817107; doi:10.1371/journal.pone.0079841)
Supplement: Table S1 — List of primers sequence used. (DOC) [file pone.0079841.s004.doc]

**Table S1. List of primers sequence used.**

| **Gene** | **Type** | **Sequence** | **Genebank Number** |
| --- | --- | --- | --- |
| Acc1 | Forward | GACCCAATTATGAATCGGGAGTG | NM_133360.2 |
|  | Reverse | CGCTCACCAACAGTAAGGTGG |  |
| ATGL | Forward | GGTCCTCCGAGAGATGTGC | NM_025802.3 |
|  | Reverse | TGGTTCAGTAGGCCATTCCTC |  |
| CD36 | Forward | CGCTTTCTGCGTATCGTCTG | NM_011977.3 |
|  | Reverse | GATGCACGGGATCGTGTCT |  |
| Cidea | Forward | TGACATTCATGGGATTGCAGAC | NM_007702.2 |
|  | Reverse | GGCCAGTTGTGATGACTAAGAC |  |
| COXIV | Forward | TACGCTGATCGGCGTGACTA | NM_009941.2 |
|  | Reverse | GCGGTACAACTGAACTTTCTCA |  |
| FABPpm | Forward | GGACCTCCAGATCCCATCCT | NM_010325.2 |
|  | Reverse | GGTTTTCCGTTATCATCCCGGTA |  |
| Fas | Forward | GGAGGTGGTGATAGCCGGTAT | NM_007988.3 |
|  | Reverse | TGGGTAATCCATAGAGCCCAG |  |
| FATP1 | Forward | TCTGTTCTGATTCGTGTTCGG | NM_011977.3 |
|  | Reverse | AAGATGCACGGGATCGTGTC |  |
| FATP2 | Forward | TCCTCCAAGATGTGCGGTACT | NM_011978.2 |
|  | Reverse | TAGGTGAGCGTCTCGTCTCG |  |
| FATP4 | Forward | ACTGTTCTCCAAGCTAGTGCT | NM_011989.4 |
|  | Reverse | GATGAAGACCCGGATGAAACG |  |
| FATP5 | Forward | CTACGCTGGCTGCATATAGATG | NM_009512.2 |
|  | Reverse | CCACAAAGGTCTCTGGAGGAT |  |
| G6Pase | Forward | CGACTCGCTATCTCCAAGTGA | NM_008061.3 |
|  | Reverse | GTTGAACCAGTCTCCGACCA |  |
| Hsl | Forward | GATGTCACAGTCAATGGAGACAC | NM_001039507.1 |
|  | Reverse | GGTGAAACCCCTCAGGGAAAG |  |
| MGL | Forward | CAGAGAGGCCAACCTACTTTTC | NM_001166251.1 |
|  | Reverse | ATGCGCCCCAAGGTCATATTT |  |
| PEPCK | Forward | CTGCATAACGGTCTGGACTTC | NM_011044.2 |
|  | Reverse | CAGCAACTGCCCGTACTCC |  |
| PGC1α | Forward | TATGGAGTGACATAGAGTGTGCT | NM_008904.1 |
|  | Reverse | CCACTTCAATCCACCCAGAAAG |  |
| Pparα | Forward | AGAGCCCCATCTGTCCTCTC | NM_011144.3 |
|  | Reverse | ACTGGTAGTCTGCAAAACCAAA |  |
| Scd1 | Forward | TTCTTGCGATACACTCTGGTGC | NM_009127.3 |
|  | Reverse | CGGGATTGAATGTTCTTGTCGT |  |
| Srebp1c | Forward | GCAGCCACCATCTAGCCTG | NM_011480.2 |
|  | Reverse | CAGCAGTGAGTCTGCCTTGAT |  |
