# Supplementary material for: Keap1-Knockdown Decreases Fasting-Induced Fatty Liver via Altered Lipid Metabolism and Decreased Fatty Acid Mobilization from Adipose Tissue
Source: PLoS One. 2013 Nov 4;8(11):e79841. doi: 10.1371/journal.pone.0079841 (PMC3817107; doi:10.1371/journal.pone.0079841)
Supplement: Table S2 — Serum cytokine content in C57BL/6 and Keap1-KD mice after 24 hours fasting. (DOCX) [file pone.0079841.s005.docx]

**Suppl Table 2. Serum cytokine content in C57BL/6 and Keap1-KD mice after 24 hours fasting**

|  | **C57BL/6** | | **Keap1-KD** | |
| --- | --- | --- | --- | --- |
|  | **Fed** | **Fasted** | **Fed** | **Fasted** |
| Glucagon, pg/ml | 9.91±4.51 | 10.20±3.57 | n.d. | n.d. |
| Resistin, ng/ml | 7661±1796 | 4544±624 | 4767±526 | 2741±287^#,§^ |
| IL-6, pg/ml | 147.54±32.56 | 50.04±20.68^*^ | 115.55±20.11 | 43.82±9.91^#^ |
| TNFα, pg/ml | 19.49±2.98 | n.d. | 38.18±16.03 | n.d. |

Fifteen- to sixteen-week-old mice C57BL/6 (WT) and Keap1-KD (KD) mice were food withhold for 24 hrs. Serum cytokines content were measured by using a Millliplex MAP kit. *, P<0.05, WT-Fasted compared with WT-Fed mice; $, P<0.05, KD-Fed compared with WT-Fed mice; #, P<0.05, KD-Fasted compared with KD-Fed mice; §, P<0.05, KD-Fasted compared with WT-Fasted mice. n.d., not detectable.
